# Digital Transformational Leadership and Coordinated Care in European Primary Healthcare: The Mediating Role of Patient Agility

**DOI:** 10.2147/JHL.S623636

**Published:** 2026-09-18

**Authors:** Liliana Hawrysz, Magdalena Kludacz-Alessandri, Wioletta Pomaranik

**Affiliations:** 1 Faculty of Management, Department of Organization and Management, Wroclaw University of Science and Technology, Wroclaw, Poland; 2 College of Economics and Social Sciences, Warsaw University of Technology, Plock, Poland

**Keywords:** digital leadership, patient responsiveness, care coordination, healthcare management

## Abstract

**Purpose:**

This study aims to examine how digital transformational leadership (DTL) is associated with coordinated care (CR) in primary healthcare organizations, with particular focus on the mediating role of patient agility (PA). By integrating the dynamic capabilities perspective, the resource-based view, and socio-technical systems theory, the study seeks to explain how leadership is associated with coordination outcomes in complex healthcare environments.

**Patients and Methods:**

Data were collected from a cross-national sample of 1,208 primary care managers in Sweden, Italy, Spain, and Germany using a structured self- reported online survey. The constructs were measured using validated multi-item scales and analyzed using structural equation modeling (SEM) in IBM SPSS AMOS. Reliability and validity were assessed through Cronbach’s alpha, composite reliability (CR), average variance extracted (AVE), and the heterotrait–monotrait ratio (HTMT).

**Results:**

The findings indicate that DTL is positive associated with patient agility (β = 0.733, p < 0.001), which in turn is positively associated with coordinated care (β = 0.761, p < 0.001). While the direct effect of DTL on coordinated care is statistically significant but weaker (β = 0.163, p < 0.01), patient agility partially mediates this relationship (β = 0.558, p < 0.001). The model demonstrates substantial explanatory power, accounting for 78.7% of the variance in coordinated care.

**Conclusion:**

The results suggest that digital transformational leadership is associated with coordinated care primarily through the development of patient agility as a dynamic organizational capability. The study highlights the importance of moving beyond technology adoption toward capability development in digital transformation initiatives. By identifying patient agility as a key mediating mechanism, the study offers theoretical and practical insights into how leadership can enhance coordination processes in primary healthcare.

## Introduction

The increasing complexity of healthcare systems, resulting from multimorbidity, fragmentation of services, and the need for collaboration among multiple entities, has intensified the importance of effective care coordination (CR), understood as the deliberate organization of care activities and the effective sharing of information among all participants involved in the care process.^1–3^

In such a context, healthcare organizations are required not only to adopt digital technologies but also to develop higher-order capabilities that enable them to continuously recognize changing patient needs and coordinate timely responses across multiple providers. These capabilities are captured by the concept of patient agility (PA), which in the healthcare information systems literature is defined as an organization’s ability to sense and respond to patient needs.^4^ While patient agility builds upon the broader concept of organizational agility developed in the dynamic capabilities literature, it represents a distinct healthcare-specific capability. Organizational agility generally refers to an organization’s ability to sense and respond to environmental changes such as market dynamics, technological developments, or competitive pressures. In contrast, patient agility narrows this perspective to the clinical context by emphasizing the continuous identification, interpretation, and response to individual patient needs throughout the care process. Rather than focusing on organizational adaptation in general, patient agility reflects a patient-centered dynamic capability that enables healthcare organizations to align clinical and organizational processes with evolving patient conditions and expectations. This distinction is particularly important in primary healthcare, where effective care coordination depends on continuously recognizing changes in patients’ health status, sharing relevant information among multiple providers, and adapting care plans accordingly. Organizations with higher patient agility are expected to detect emerging patient needs earlier, facilitate timely communication across professional boundaries, and coordinate appropriate responses, thereby reducing fragmentation of care and improving continuity, integration, and responsiveness of healthcare services.

Although prior research has primarily focused on technological antecedents of patient agility, such as IT ambidexterity, digital capabilities, and knowledge processes, the role of leadership as a microfoundation of these capabilities remains underexplored, particularly in healthcare settings.

In this context, the concept of digital transformational leadership (DTL) provides an important theoretical perspective, extending traditional transformational leadership by incorporating leaders’ ability to guide organizations through digital transformation processes, promote innovation, and foster adaptability in dynamic technological environments.^5^

Despite the growing interest in digital transformation in healthcare, limited empirical evidence exists on how digital transformational leadership translates into specific organizational capabilities and, ultimately, into improved care coordination outcomes.

Although prior studies have examined the relationship between digital transformation and organizational agility, the concept of “patient agility” remains insufficiently conceptualized and empirically validated in the healthcare literature. Existing research has predominantly focused on general organizational agility, while its patient-centered dimension, reflecting the ability to sense and respond to patient needs, has received limited attention.^5–7^ Moreover, recent research has rarely examined whether patient agility mediates the relationship between digital transformational leadership and care coordination. While digital leadership and organizational agility have been investigated separately, empirical studies integrating these constructs within a single theoretical framework remain scarce.

Furthermore, the mechanisms linking agility to concrete care coordination outcomes remain largely conceptual or have been explored primarily through qualitative approaches. Consequently, empirical evidence explaining how digital transformational leadership enhances care coordination by developing patient agility is still limited. In particular, previous studies have rarely incorporated measurable process-oriented indicators of coordination, such as cross-provider handoffs or the timing and integration of services.^8^

As a result, while digital transformational leadership is recognized as a driver of digital capabilities and organizational agility, its role in fostering patient agility and translating this capability into improved care coordination remains insufficiently understood. To better explain these relationships, this study integrates three complementary theoretical perspectives. Resource- Resource-Based View (RBV) provides the foundation for understanding digital transformational leadership as a strategic managerial capability that enables healthcare organizations to effectively leverage valuable digital resources. Dynamic Capabilities Theory extends this perspective by explaining how leadership enables organizations to develop higher-order capabilities, particularly patient agility, which allows healthcare organizations to continuously sense and respond to changing patient needs. Finally, Socio-Technical Systems Theory emphasizes that improvements in care coordination emerge not from technology alone but from the effective alignment of technological capabilities, organizational processes, leadership practices, and healthcare professionals. Together, these perspectives provide a comprehensive framework explaining how digital transformational leadership fosters patient agility and, ultimately, contributes to improved care coordination.

To address this gap, the aim of this study is to examine the relationships between digital transformational leadership (DTL), patient agility (PA), and care coordination (CR) within healthcare organizations. Specifically, the study investigates whether patient agility mediates the relationship between DTL and care coordination and seeks to answer the following research question: How does digital transformational leadership influence care coordination through the development of patient agility?

In doing so, and by integrating the Resource-Based View, Dynamic Capabilities Theory, and Socio-Technical Systems Theory,^9^ this study contributes to the literature in three ways. First, it extends the emerging literature on patient agility by positioning digital transformational leadership as one of its key organizational antecedents, a relationship that has received limited empirical attention in healthcare. Second, it provides empirical evidence demonstrating the mediating role of patient agility in translating digital transformational leadership into improved care coordination. Third, it enriches the healthcare management literature by linking digital transformation leadership with care coordination, a critical outcome in complex healthcare systems.

The proposed research model is empirically tested using structural equation modeling (SEM) on survey data collected from healthcare managers. The results provide insights into the role of leadership in developing agile capabilities and improving coordination processes in digitally transforming healthcare organizations.

## Literature Review and Hypotheses Development

Patient agility represents a higher-order capability composed of two complementary components: the ability to identify and anticipate patient needs and the ability to rapidly adapt care processes and services in response to those needs. Consistent with the dynamic capabilities perspective, patient agility reflects an organization’s ability to integrate, build, and reconfigure resources in response to changing environmental conditions.^10^

Previous research has primarily focused on technological antecedents of patient agility, such as IT ambidexterity, digital capabilities, and knowledge processes,^4^ while the role of leadership as a microfoundation of these capabilities remains underexplored.

Empirical studies indicate that DTL enhances organizational agility and adaptive capabilities that support digital transformation.^5^,^6^ In particular, digitally transformational leaders foster sensing capabilities by promoting data-driven decision-making and the use of digital technologies for identifying patient needs, while simultaneously enabling responding capabilities through process redesign, cross-functional collaboration, and organizational flexibility.

From the resource-based view (RBV), DTL can be understood as a managerial capability that facilitates the orchestration of key digital resources, such as clinical data, system interoperability, and staff competencies.^11^,^12^ Integrating the RBV and dynamic capabilities perspectives, DTL can be viewed as a key enabler that activates and aligns organizational resources to develop patient agility.

This relationship is also supported by the broader healthcare leadership literature, which indicates that transformational leadership fosters better collaboration, work environments, and patient outcomes.^13^ Importantly, while prior research has largely emphasized technological drivers of agility, this perspective overlooks the role of leadership in activating and directing these capabilities, suggesting that digital transformational leadership may serve as a critical but underexplored mechanism in shaping patient agility.^14^ Thus, building on the dynamic capabilities and RBV perspectives, as well as prior empirical findings, digital transformational leadership is expected to play a critical role in developing patient agility. Therefore, the following hypothesis is proposed:

**H1**: Digital transformational leadership positively influences patient agility.

Patient agility also has direct implications for coordinated care. In increasingly complex healthcare environments, organizations must not only possess information but also be able to interpret and utilize it effectively in decision-making processes.^4^,^15^ According to the logic of dynamic capabilities, organizations capable of rapidly detecting changes and responding adaptively are better equipped to operate in complex and uncertain environments.^10^ Patient agility enables early identification of patient needs and adaptation of care processes, thereby facilitating coordination among different actors within the healthcare system, particularly in the case of patients requiring multidisciplinary care.^16^ In particular, sensing capabilities support the timely identification of patient needs across organizational boundaries, while responding capabilities enable the alignment and synchronization of care activities, reducing fragmentation and improving continuity of care.

Research indicates that effective care coordination depends on information flow, collaboration, and organizational adaptability.^4^,^17^,^18^ Agile organizations are more likely to engage in knowledge sharing, inter-unit collaboration, and flexible process adjustments, which support service integration and improve communication among healthcare providers.^15^,^19^,^20^ However, the mere availability of information does not guarantee effective coordination;^21^ rather, organizations must possess the capability to translate information into coordinated actions, highlighting the critical role of patient agility in bridging this gap.^18^ As a result, higher levels of patient agility enhance an organization’s ability to deliver coordinated care.

Therefore, the following hypothesis is proposed:

**H2**: Patient agility positively influences coordinated care.

At the same time, from the perspective of socio-technical systems, the effectiveness of care coordination depends on the alignment of technology, people, processes, and organizational rules.^12^ DTL facilitates this alignment by building a shared vision of transformation, strengthening collaboration, and supporting the integration of technology into clinical practice. The literature indicates that leadership plays a crucial role in shaping conditions that foster collaboration, trust, and effective communication, which are fundamental to care coordination.^22–24^ Furthermore, leaders support knowledge flows and the integration of information across entities, which are essential for effective coordination.^25^,^26^ In the context of digital transformation, leadership also plays a key role in developing organizational capabilities for the effective use of digital technologies and data-driven systems, thereby supporting collaboration and integration of care.^19^,^27^ Studies also emphasize the importance of leaders’ digital competencies in managing complex care processes and coordinating activities in data-driven environments.^28^,^29^ Importantly, beyond its indirect influence through organizational capabilities such as patient agility, DTL may also exert a direct effect on care coordination by shaping governance structures, standardizing processes, and enabling cross-organizational alignment.^30^ Thus, digitally transformational leaders not only foster capabilities that enhance coordination but may also directly influence coordination mechanisms through the design of processes, communication structures, and coordination routines. Therefore, DTL is also expected to have a direct impact on coordinated care.

Therefore, the following hypothesis is proposed:

**H3**: Digital transformational leadership positively influences coordinated care.

Integrating the above arguments, it can be observed that DTL creates conditions for the development of digital resources and capabilities; however, its impact on organizational outcomes is realized primarily through more proximal operational capabilities. From the perspective of dynamic capabilities and the resource-based view, leadership can be conceptualized as a distal enabler,^31^ whereas organizational outcomes emerge through proximal capabilities that directly shape organizational processes and actions.^32^ In this context, patient agility represents such a proximal capability, translating leadership-driven resources and transformation initiatives into concrete organizational behaviors.

Patient agility enables the transformation of digital and organizational potential into concrete actions that support care coordination, such as effective information flow, rapid response, and integration of services across entities.^33^,^34^ More specifically, by enhancing sensing capabilities (identification and anticipation of patient needs) and responding capabilities (adaptation and coordination of care processes), patient agility provides the mechanism through which leadership influences coordination outcomes. Therefore, rather than exerting its influence solely directly, DTL is expected to affect care coordination primarily through the development of patient agility.

Therefore, the following hypothesis is proposed:

**H4**: Patient agility mediates the relationship between digital transformational leadership and coordinated care.

## Material and Methods

This study aims to examine the impact of digital transformational leadership (DTL) on patient agility (PA) and, consequently, on coordinated care (CR) among managers of primary care facilities.

Patient agility is operationalized as a unidimensional construct reflecting the organization’s overall ability to sense and respond to patient needs. Although conceptually encompassing both sensing capabilities (SENSE) and responding capabilities (RESP), confirmatory factor analysis (CFA) results indicated that all related indicators load onto a single latent factor. Alternative model specifications distinguishing these dimensions were tested; however, the unidimensional structure demonstrated superior fit, supporting its treatment as a single construct in this study.

Similarly, coordinated care is operationalized as a unidimensional construct reflecting the overall level of coordination across different domains of care. Although it includes internal coordination (CR-C), coordination with external providers (CR-P), and coordination with community resources (CR-R), CFA results confirmed that all indicators load onto a single latent factor, supporting a parsimonious representation of the construct.

**Figure 1:**
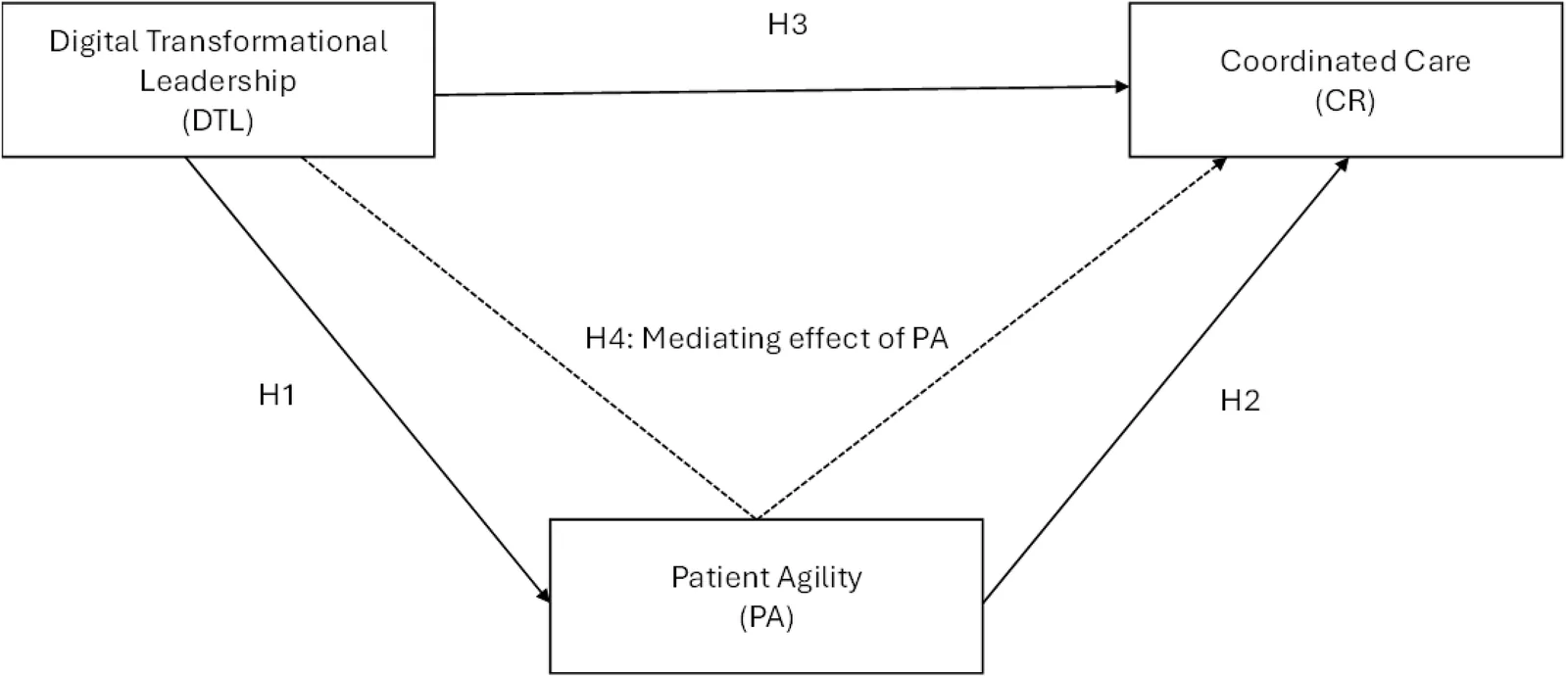
The conceptual research model.

The conceptual research model and the hypothesized relationships between the constructs are presented in Figure 1.

### Sampling

The study draws on a cross-national sample of 1,208 primary care managers working in primary care organizations in Sweden (303), Italy (302), Spain (301), and Germany (302). Data were collected in January 2026 using a web-based survey approach (CAWI). Participants were recruited through commercial non-probability online research panels administered by a specialized survey provider. The panels comprise pre-screened individuals who have agreed to participate in online surveys.

The inclusion criterion was holding a managerial position in a primary care organization and to be directly involved in organizational and/or digital decision-making processes. Eligibility was assessed through screening questions administered at the beginning of the survey, and respondents who did not meet this criterion were screened out.

Country-specific quotas were established to obtain approximately equal numbers of completed responses from each of the four countries. Data collection continued until the prespecified number of complete and valid responses was obtained in each country, ensuring comparability across national subsamples. Because recruitment and the distribution of survey invitations were managed by the panel provider, the researchers did not have access to the total number of invitations sent or complete invitation-level disposition data. Consequently, an invitation-based response rate could not be calculated.

The final sample size is considered adequate for structural equation modeling (SEM), exceeding commonly recommended thresholds for both minimum sample size and the ratio of observations to estimated parameters.^35^,^36^

Responses were provided anonymously to the research team, and confidentiality was maintained throughout the study. To ensure data quality, several control procedures were applied by the panel provider, including respondent verification, screening for eligibility, monitoring of completion time, and checks for response consistency. Only responses that met predefined quality criteria were included in the final dataset.

Given the use of commercial non-probability online panels, potential limitations related to self-selection and coverage bias should be acknowledged. However, the use of country-specific quotas, eligibility screening, and quality-control procedures contributed to the consistency and comparability of the national subsamples. The socio-demographic characteristics of the respondents are presented in Table 1

**Table 1:** Demographic and Socio-Economic Characteristics of Respondents

Characteristics	Classification	N	%
Gender	Female	636	52.65
	Male	552	45.69
	Other	10	0.83
	No answer	10	0.83
Age	18–24	101	8.36
	25–34	331	27.40
	36–44	348	28.81
	46–54	257	21.27
	56–64	139	11.51
	65+	28	2.32
	No answer	4	0.33
Educational background	Medical background (eg, doctors, nurses, midwives)	593	49.1
	Business, management and administrative background	485	40.1
	Other	51	4.2
	Two educational backgrounds	68	5.6
	Three educational backgrounds	2	0.2
	No educational background reported	9	0.7

The sample was relatively balanced in terms of gender, with 52.65% female and 45.69% male respondents, while only a marginal proportion identified as other or did not provide an answer (each 0.83%). In terms of age distribution, the largest groups were respondents aged 36–44 years (28.81%) and 25–34 years (27.40%), followed by those aged 46 −54 (21.27%). Younger (18–24) and older respondents (56+) were less represented, accounting for 8.36% and 13.83% of the sample, respectively Regarding educational background, 49.1% of respondents reported only a medical background (eg, physicians, nurses, and midwives), 40.1% reported only a business, management, or administrative background, and 4.2% reported only other educational backgrounds. In addition, 5.6% of respondents reported two educational backgrounds and 0.2% reported three educational backgrounds, whereas 0.7% did not report their educational background. Overall, the sample reflects a diverse group of primary care managers with both clinical and managerial expertise, which is particularly relevant for examining digital transformation and coordination processes in healthcare settings.

## Measures

The survey instrument consisted of two sections. The first section collected respondents’ socio-demographic and professional characteristics, including gender, age, and educational background. The second section included measurement items for the three theoretical constructs used in the research model: digital transformational leadership (DTL), patient agility (PA), and coordinated care (CR).

All constructs were measured using previously validated scales adapted to the context of primary care facilities and the management of patients with multimorbidity. The wording of the items was adjusted to reflect the organizational and clinical context of primary healthcare. Given the cross-national nature of the study, the questionnaire was translated using a back-translation procedure to ensure linguistic and conceptual equivalence across countries. All items were assessed using a five-point Likert scale ranging from 1 = strongly disagree to 5 = strongly agree.

Digital transformational leadership (DTL) was measured using six items adapted from prior research. The scale builds on the concept of transformational leadership^37^,^38^ and has been extended to the digital transformation context in more recent studies.^39^ The items capture leaders’ ability to articulate a digital transformation vision, motivate employees toward digital goals, and foster engagement with digital transformation initiatives. In this study, DTL is conceptualized as the leadership capability to mobilize organizational members in support of digital transformation within primary care settings.

Patient agility refers to an organization’s ability to effectively identify and respond to patient needs. The construct is grounded in the dynamic capabilities perspective and has originally been conceptualized as a higher-order capability composed of patient sensing and patient responding dimensions.^40^,^41^ In healthcare research, this concept has been adapted to capture how healthcare organizations recognize and react to patient needs in complex and dynamic care environments^4^,^42^ In the present study, patient agility was measured using items adapted from the original scale developed by Roberts and Grover.^41^ Although the original conceptualization distinguishes sensing and responding capabilities, confirmatory factor analysis (CFA) results indicated that all items loaded significantly onto a single latent factor.

To ensure robustness, alternative model specifications distinguishing sensing and responding as separate dimensions were also tested; however, the unidimensional model demonstrated superior model fit and greater parsimony. Therefore, patient agility was operationalized as a unidimensional construct reflecting the overall ability of primary care facilities to sense and respond to patient needs. This operationalization is consistent with prior research treating agility as an integrated capability that captures the joint effect of sensing and responding processes.

Coordinated care (CR) was measured using selected items adapted from the Provider and Staff Perceptions of Integrated Care (PSPIC) survey.^43^ The original PSPIC instrument was developed to capture providers’ and staff perceptions of integrated care. The PSPIC includes items related to care coordination within the care team, coordination with external providers, and coordination with community resources. In the present study, coordinated care was operationalized as a unidimensional construct, because CFA results indicated that all coordination-related indicators loaded onto a single latent factor.

Similarly, alternative multidimensional structures were tested; however, the single-factor model provided a more parsimonious and empirically supported representation of the construct.

Accordingly, CR represents the overall perceived level of coordination in the care of patients with multimorbidity across internal teams, external providers, and community-based resources.

This approach aligns with prior studies that conceptualize care coordination as a holistic organizational capability rather than a set of independent dimensions. The complete list of measurement items is provided in Appendix A.

### Data Analysis

The data were analyzed using structural equation modeling (SEM). The analysis was conducted in two stages. First, confirmatory factor analysis (CFA) was performed to assess the measurement model. Reliability and validity were evaluated using Cronbach’s alpha, composite reliability (CR), average variance extracted (AVE), and the heterotrait–monotrait ratio (HTMT).

In the second stage, the structural model was estimated to test the hypothesized relationships between the constructs. Model fit was assessed using commonly recommended indices, including χ^2^/df, CFI, TLI, and RMSEA. The statistical significance of the indirect association between DTL and CR through PA was assessed using 2,000 bootstrap samples and 95% bootstrap confidence intervals. An indirect association was considered statistically significant when the 95% confidence interval did not include zero.

Missing values accounted for approximately 1.15% of all responses across the 25 measurement items, with item-level missingness ranging from approximately 0.4% to 2.1%. Item-mean imputation was used in the primary analyses to retain the full analytical sample and enable bootstrap estimation of the indirect association in AMOS. To assess the robustness of the findings to the missing-data procedure, the principal structural model was additionally re-estimated using full information maximum likelihood (FIML) on the original incomplete dataset.

Because all constructs were measured using the same questionnaire and respondent source, potential common method bias was assessed using two complementary procedures. First, Harman’s single-factor test was conducted by entering all 25 measurement items into an unrotated principal component analysis and examining the proportion of variance explained by the first extracted component. Second, an unmeasured common latent factor approach was applied in AMOS. In this model, all measurement items were allowed to load simultaneously on their theoretically specified constructs and on an orthogonal common latent factor. The variance of the common latent factor was fixed to 1 for model identification. The fit of the model containing the common latent factor was compared with that of the baseline model without the method factor. The structural paths were subsequently re-estimated while controlling for the common latent factor to assess whether shared method variance materially affected the direction or statistical significance of the hypothesized relationships.

Given the cross-national design of the study, measurement invariance across Sweden, Germany, Italy, and Spain was assessed using multi-group confirmatory factor analysis. Configural invariance was examined by estimating the same factor structure across countries while allowing the model parameters to vary freely. Metric invariance was then tested by constraining the factor loadings to equality across groups, followed by scalar invariance testing, in which both factor loadings and item intercepts were constrained to equality. Model comparisons were based primarily on changes in the comparative fit index (CFI) and root mean square error of approximation (RMSEA). Measurement invariance was considered supported when the decrease in CFI did not exceed 0.010 and the increase in RMSEA did not exceed 0.015. After establishing scalar invariance, multi-group structural equation modeling was conducted to assess whether the hypothesized structural paths differed across the four countries. An unconstrained structural model was compared with a model in which the paths from DTL to PA, from PA to CR, and from DTL to CR were constrained to equality across countries.

All statistical analyses were performed using IBM SPSS Statistics (version 11) and AMOS (version 31).

## Results

### Measurement Model Validity

The measurement model was assessed prior to testing the structural relationships to ensure the reliability and validity of the constructs.

Internal consistency reliability was first evaluated using Cronbach’s alpha. The results indicate satisfactory to good reliability across all constructs. Digital transformation leadership (DTL) achieved a Cronbach’s alpha of 0.748, while patient agility (PA) and coordinated care (CR) demonstrated higher reliability levels, with values of 0.816 and 0.807, respectively. All values exceed the recommended threshold of 0.70, indicating acceptable internal consistency. Item–total correlations were above the minimum acceptable level, and the “alpha if item deleted” analysis showed no substantial improvement in reliability upon removal of any individual item, supporting the internal consistency of the scales.

Convergent validity was assessed using standardized factor loadings, composite reliability (CR), and average variance extracted (AVE). As shown in Table 2, AVE values ranged from 0.303 to 0.332 and were below the conventional threshold of 0.50. However, all constructs demonstrated acceptable composite reliability (CR > 0.70). According to Hair et al (2019),^44^ this combination indicates adequate convergent validity when supported by satisfactory factor loadings and composite reliability.

**Table 2:** Measurement Model Assessment

Construct	Item	Standardized Loading	Cronbach’s α	CR	AVE
Digital Transformational Leadership (DTL)	DTL1	0.57	0.748	0.748	0.332
	DTL2	0.61			
	DTL3	0.59			
	DTL4	0.57			
	DTL5	0.54			
	DTL6	0.58			
Patient Agility (PA)	SENSE1	0.527	0.816	0.812	0.303
	SENSE2	0.477			
	SENSE3	0.514			
	SENSE4	0.607			
	SENSE5	0.482			
	RESPOND1	0.599			
	RESPOND2	0.557			
	RESPOND3	0.596			
	RESPOND4	0.568			
	RESPOND5	0.562			
Coordinated Care (CR)	CR_C1	0.553	0.807	0.801	0.310
	CR_C2	0.573			
	CR_C3	0.565			
	CR_P1	0.592			
	CR_P2	0.560			
	CR_P3	0.502			
	CR_R1	0.520			
	CR_R2	0.581			
	CR_R3	0.560			

Discriminant validity was assessed using the heterotrait–monotrait ratio (HTMT), which is considered a robust criterion for evaluating discriminant validity. As presented in Table 3, all HTMT values were below the conservative threshold of 0.85, providing strong evidence of discriminant validity between the constructs.

**Table 3:** HTMT Results for Discriminant Validity

Construct	DTL	PA	CR
Digital Transformational Leadership (DTL)	–	0.74	0.62
Patient Agility (PA)	0.74	–	0.76
Coordinated Care (CR)	0.62	0.76	–

**Notes**: HTMT values below 0.85 indicate satisfactory discriminant validity.

Overall, the measurement model demonstrates satisfactory reliability, adequate convergent validity, and strong discriminant validity, supporting its use in the structural model.

These results confirm that the constructs are empirically distinct and capture different theoretical concepts.

#### Common Method Bias Assessment

Because all constructs were measured using the same survey instrument and respondent source, potential common method bias was assessed using two complementary procedures. First, Harman’s single-factor test was conducted by entering all 25 measurement items into an unrotated principal component analysis. Three components with eigenvalues greater than 1 were extracted, jointly accounting for 41.116% of the total variance. The first component explained 31.129% of the variance, which was below the conventional threshold of 50%. This result indicates that no single factor dominated the covariance among the survey items.

A more stringent assessment was subsequently conducted using an unmeasured common latent factor (CLF) approach. The baseline model without the common method factor demonstrated a good fit to the data (χ^2^ = 443.176, df = 272, χ^2^/df = 1.629, CFI = 0.976, TLI = 0.974, RMSEA = 0.023). The model including the common latent factor also demonstrated a good fit (χ^2^ = 381.931, df = 247, χ^2^/df = 1.546, CFI = 0.981, TLI = 0.977, RMSEA = 0.021). Adding the common latent factor significantly improved model fit, Δχ^2^(25) = 61.245, p < 0.001, although the changes in CFI and RMSEA were relatively small (ΔCFI = 0.005; ΔRMSEA = −0.002). These findings indicate the presence of some shared method-related variance.

To assess whether this shared variance accounted for the substantive findings, the structural relationships were re-estimated while controlling for the common latent factor. The relationships between DTL and PA (β = 0.537, p = 0.014), PA and CR (β = 0.572, p < 0.001), and DTL and CR (β = 0.204, p < 0.001) remained positive and statistically significant. Although the magnitudes of the DTL–PA and PA–CR relationships were attenuated compared with the baseline model, their direction and statistical significance remained unchanged. Taken together, the results of Harman’s single-factor test and the common latent factor analysis suggest that common method variance accounted for part of the shared variance among the measures but did not fully explain the principal structural relationships.

#### Cross-National Measurement Invariance and Multi-Group Analysis

Given the cross-national design of the study, measurement invariance across Sweden, Germany, Italy, and Spain was assessed before comparing the structural relationships. Configural, metric, and scalar invariance were tested sequentially using multi-group confirmatory factor analysis.

The configural model demonstrated an acceptable fit to the data (χ^2^ = 1624.925, df = 1088, CFI = 0.930, TLI = 0.923, RMSEA = 0.020), supporting the equivalence of the general factor structure across the four countries. Constraining the factor loadings to equality did not result in a deterioration in model fit (metric model: χ^2^ = 1673.963, df = 1154, CFI = 0.933, TLI = 0.930, RMSEA = 0.019; ΔCFI = 0.003; ΔRMSEA = −0.001). Metric invariance was therefore supported.

The scalar invariance model, in which both factor loadings and item intercepts were constrained to equality, also demonstrated an acceptable fit (χ^2^ = 1750.928, df = 1229, CFI = 0.932, TLI = 0.934, RMSEA = 0.019). Compared with the metric model, the scalar constraints did not meaningfully worsen model fit (ΔCFI = −0.001; ΔRMSEA = 0.000), supporting scalar measurement invariance. These findings indicate that the constructs were measured in a sufficiently comparable manner across the four national samples.

After establishing scalar invariance, multi-group structural equation modeling was used to examine whether the hypothesized structural relationships differed across countries. An unconstrained structural model, in which the paths from DTL to PA, from PA to CR, and from DTL to CR were estimated separately in each country, was compared with a model in which all three structural paths were constrained to equality across countries.

The constrained model demonstrated an acceptable fit to the data (χ^2^ = 1764.643, df = 1238, CFI = 0.932, TLI = 0.934, RMSEA = 0.019). Constraining the structural paths to equality did not significantly worsen model fit, Δχ^2^(9) = 13.715, p = 0.133, and no changes were observed in CFI or RMSEA (ΔCFI = 0.000; ΔRMSEA = 0.000). These results indicate that the structural relationships among digital transformational leadership, patient agility, and coordinated care were broadly equivalent across Sweden, Germany, Italy, and Spain. The differences visible in the country-specific standardized coefficients were not statistically sufficient to reject the equality of the structural paths across countries. Accordingly, the pooled structural model was retained for hypothesis testing. A summary of the measurement invariance and multi-group structural analyses is presented in Table 4.

**Table 4:** Cross-National Measurement Invariance and Structural Path Equivalence

Model	χ^2^	df	χ^2^/df	CFI	TLI	RMSEA	ΔCFI	ΔRMSEA
Configural invariance	1624.925	1088	1.493	0.930	0.923	0.020	–	–
Metric invariance	1673.963	1154	1.451	0.933	0.930	0.019	0.003	−0.001
Scalar invariance	1750.928	1229	1.425	0.932	0.934	0.019	−0.001	0.000
Structural paths unconstrained	1750.928	1229	1.425	0.932	0.934	0.019	–	–
Structural paths constrained	1764.643	1238	1.425	0.932	0.934	0.019	0.000	0.000

**Notes**: Changes in fit indices for the metric model were calculated relative to the configural model, changes for the scalar model relative to the metric model, and changes for the constrained structural model relative to the unconstrained structural model. The comparison between the unconstrained and constrained structural models was non-significant: Δχ^2^(9) = 13.715, p = 0.133.

#### Model Fit Assessment

Because the multi-group analysis supported the equivalence of the structural relationships across the four countries, the pooled structural model was used to test the study hypotheses. The structural model included digital transformational leadership (DTL) as the independent variable, patient agility (PA) as the mediating variable, and coordinated care (CR) as the dependent variable. The final model consisted of 25 observed variables and three latent variables.

The model achieved an excellent fit to the data (χ^2^ = 443.176, df = 272, p < 0.001, χ^2^/df = 1.629). Incremental fit indices further confirmed the model’s adequacy, with CFI = 0.976, TLI = 0.974, IFI = 0.976, and NFI = 0.941. The RMSEA value was 0.023, with a 90% confidence interval of 0.019–0.027 and PCLOSE = 1.000, indicating excellent approximate fit. Parsimony-adjusted indices were also satisfactory (PNFI = 0.853; PCFI = 0.885).

Overall, these results indicate that the proposed model provides a very good representation of the observed data and can be considered appropriate for testing the hypothesized relationships.

A summary of the model-fit indices is presented in Table 5.

**Table 5:** Summary of Model-Fit Indices for Structural Model

Model-Fit Index	χ^2^/df	RMSEA	CFI	NFI	TLI	IFI	PNFI	PCFI	PClose
Results	1.629	0.023	0.976	0.941	0.974	0.976	0.853	0.885	1.000

#### Structural Model and Hypotheses Testing

**Figure 2:**
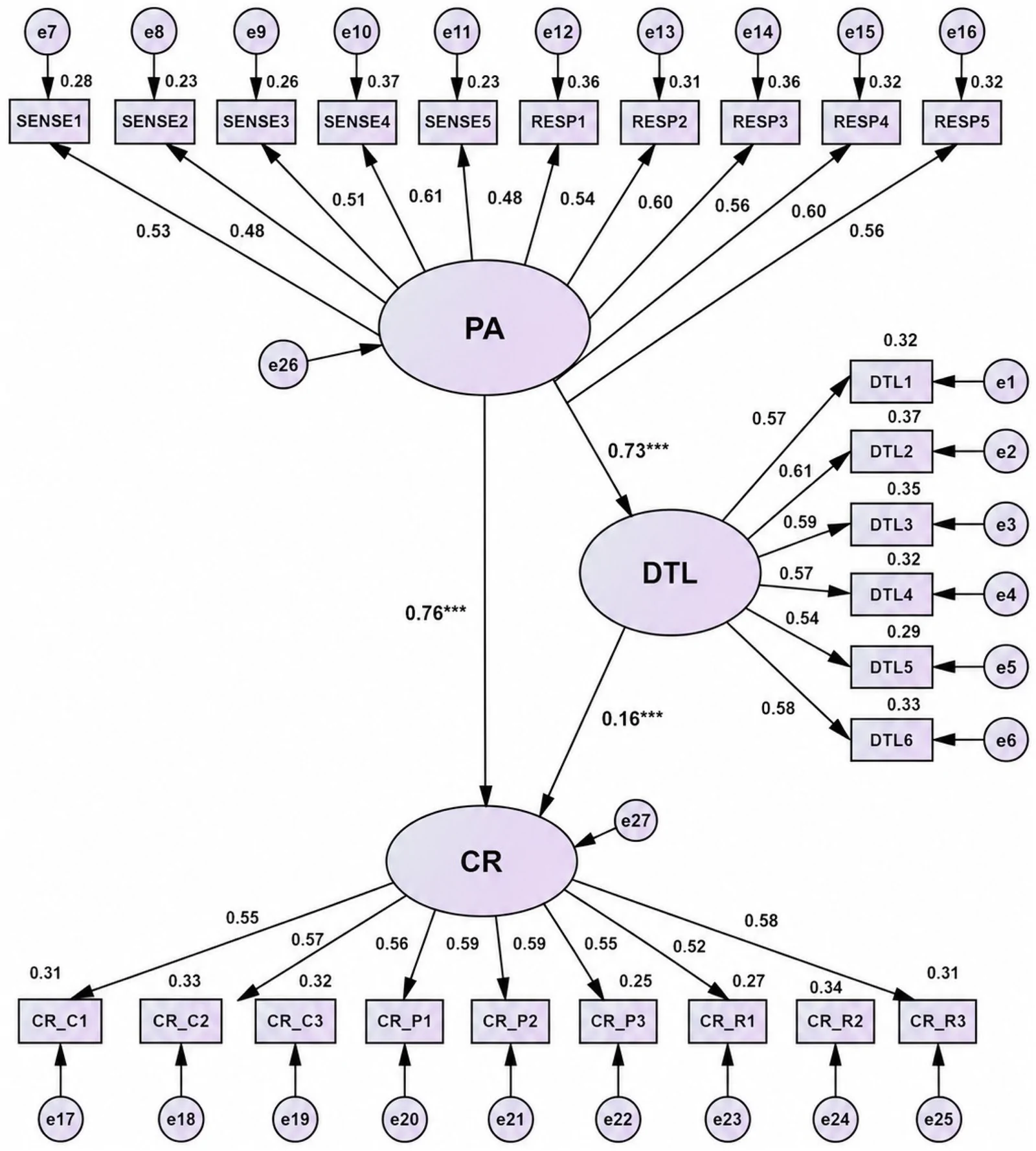
Final structural equation model with standardized estimates.

**Note: Values represent standardized path coefficients. *p < 0.05; **p < 0.01; *p < 0.001.

Following the establishment of the measurement model’s reliability and validity, the structural model was evaluated to test the hypothesized relationships among the constructs. The assessment focused on the significance and strength of the standardized path coefficients (β), as well as the explanatory power of the model, assessed using the coefficient of determination (R^2^) for the endogenous variables. According to Hair et al (2019),^44^ R^2^ values of 0.75, 0.50, and 0.25 can be considered substantial, moderate, and weak, respectively. The standardized path coefficients and their statistical significance are presented in Figure 2 and Table 6.

**Table 6:** Hypotheses Testing Results (N = 1208)

Hypothesis	Path	Path Coefficient (*β)*	Hypothesis Acceptance
H1	DTL → PA	0.733***	Supported
H2	PA → CR	0.761***	Supported
H3	DTL → CR	0.163***	Supported
H4	DTL → PA → CR	0.558***	Supported (partial mediation)

**Note**: *** p < 0.001.

The results indicate that digital transformational leadership (DTL) is strongly and positively associated with patient agility (PA) (β = 0.733, p < 0.001), suggesting that higher levels of leadership engagement in digital transformation are related to a greater organizational ability to sense and respond to patient needs.

Patient agility, in turn, is strongly and positively associated with coordinated care (CR) (β = 0.761, p < 0.001), indicating that agile capabilities are related to better coordination outcomes across healthcare processes.

In addition to the indirect association, DTL also shows a statistically significant direct association with coordinated care (β = 0.163, p < 0.001), although this relationship is notably weaker than the indirect association.

The coefficient of determination (R^2^) shows that DTL explains 53.7% of the variance in patient agility, indicating a moderate-to-high level of explanatory power. Furthermore, DTL and PA jointly explain 78.7% of the variance in coordinated care, indicating substantial explanatory power.

However, this comparatively high value should be interpreted cautiously because all constructs were measured simultaneously using the same respondent source. In the common latent factor robustness model, the explained variance in coordinated care decreased to 49.4%, suggesting that shared method variance may have contributed to the magnitude of the baseline R^2^. Nevertheless, the structural relationships remained positive and statistically significant after controlling for the common method factor, indicating that they were not fully attributable to common source variance.

To further examine the mediating role of patient agility, indirect effects were assessed using bootstrapping procedures. The indirect association between DTL and coordinated care through patient agility was strong and statistically significant (β = 0.558, p < 0.001), with the bootstrap confidence interval excluding zero.

These findings provide evidence that patient agility acts as a statistically significant mediator of the association between digital transformational leadership and coordinated care. Given that both the direct and indirect associations are statistically significant, the results indicate partial statistical mediation. Notably, the indirect association is substantially stronger than the direct association, suggesting that the relationship between leadership and coordinated care is largely accounted for statistically by patient agility rather than by the direct relationship alone.

Finally, a sensitivity analysis was conducted by re-estimating the principal structural model using full information maximum likelihood (FIML) on the original incomplete dataset. The FIML model demonstrated very good fit (χ^2^ = 446.733, df = 272, χ^2^/df = 1.642, CFI = 0.976, TLI = 0.971, RMSEA = 0.023). The standardized structural coefficients were virtually unchanged: DTL was positively associated with PA (β = 0.735, p < 0.001), PA was positively associated with CR (β = 0.767, p < 0.001), and the direct association between DTL and CR remained statistically significant (β = 0.156, p = 0.001). The standardized indirect effect was also comparable with that obtained in the primary analysis (β = 0.564). These results indicate that the substantive conclusions were not materially affected by the missing-data procedure.

## Discussion

The aim of this study was to examine the statistical pathways through which digital transformational leadership is associated with coordinated care in primary healthcare organizations, with particular emphasis on the mediating role of patient agility. The findings largely support the proposed theoretical model. Positive associations were identified between digital transformational leadership and patient agility (H1) and between patient agility and coordinated care (H2). The indirect association between digital transformational leadership and coordinated care through patient agility was also statistically significant (H4). Importantly, the direct association between digital transformational leadership and coordinated care (H3) remained statistically significant, although it was notably weaker than the indirect association. These results suggest that the association between digital leadership and care coordination is largely accounted for statistically by the development of organizational capabilities rather than by direct managerial intervention alone. However, because the study used a cross-sectional design, this pattern should not be interpreted as evidence of a definitive causal mechanism.

The positive association between digital transformational leadership and patient agility is consistent with the assumptions of Dynamic Capabilities Theory. Organizations operating in complex and uncertain environments must develop the ability to integrate, build, and reconfigure resources in response to changing conditions.^10^ In the healthcare context, patient agility represents such a capability, as it encompasses both sensing identifying and anticipating patient needs and responding rapidly adapting care processes and services to those needs.^4^,^18^

The findings suggest that digital transformational leadership may function as an important organizational microfoundation of dynamic capabilities by strengthening the routines and processes from which patient agility emerges. By articulating a clear digital vision, promoting the use of digital technologies, encouraging data-driven decision-making, and fostering collaboration among healthcare professionals, leaders create conditions that support the development of sensing and responding capabilities. This finding extends previous research, which has primarily examined technological antecedents of patient agility, including IT ambidexterity, digital dynamic capability, and knowledge processes,^4^,^18^ by indicating that leadership-related practices are also closely associated with the development of this capability.

The positive association between patient agility and coordinated care reinforces the argument that dynamic capabilities are linked to observable healthcare delivery processes. This finding is consistent with prior literature indicating that effective care coordination depends not only on access to information but also on the ability of organizations to interpret, integrate, and act upon that information in a timely manner.^2–4^,^18^ In environments characterized by multimorbidity and fragmented service provision, healthcare organizations must continuously identify evolving patient needs, translate them into coordinated clinical and organizational actions, and dynamically adjust care pathways across multiple providers. Patient agility captures this organizational capacity by combining the early recognition of patient needs with flexible and timely responses.

Patient agility can therefore be understood as an enabling capability that supports information continuity, facilitates the closing of clinical loops, and strengthens the integration of services across organizational boundaries. The results are consistent with the proposition that higher-order organizational capabilities are associated with process-level outcomes in healthcare settings. They are also aligned with research on integrated care, which emphasizes the importance of collaboration, information flow, and organizational adaptability in improving care coordination.^16^,^19^,^20^ The present study extends this literature by identifying patient agility as a specific organizational capability through which these factors may be translated into more coordinated care processes.

The substantial proportion of variance explained in coordinated care in the baseline model (R^2^ = 0.787) should nevertheless be interpreted cautiously. DTL and PA are theoretically related to coordinated care, and all constructs were measured simultaneously using the same respondent source. In the common latent factor robustness model, the explained variance in coordinated care decreased to 49.4%. This reduction suggests that shared method-related variance may have contributed to the magnitude of the baseline R^2^. However, the three principal structural associations remained positive and statistically significant after controlling for the common method factor. Thus, common source variance appears to explain part, but not all, of the observed relationships. Accordingly, the baseline R^2^ should not be interpreted as evidence that DTL and PA exhaustively determine coordinated care.

The relatively weak direct association of digital transformational leadership on coordinated care carries important theoretical implications. It suggests that digital leadership is unlikely to translate automatically into improved care coordination. Even when leaders successfully promote digitalization, support innovation, and create a favorable organizational climate, these efforts may be reflected in coordination processes primarily when they are translated into more proximal operational capabilities. The observed statistical mediation pattern identifies patient agility as such a capability.

This interpretation is consistent with both Dynamic Capabilities Theory and the Resource-Based View. From an RBV perspective, the mere possession of resources or managerial mechanisms is insufficient for value creation; what matters is the existence of capabilities that enable the effective deployment of these resources.^11^ In healthcare, digital transformational leadership can be understood as a mechanism for orchestrating digital resources such as clinical data, interoperability, staff competencies, and analytical tools.^12^ However, these resources do not generate value unless they are effectively mobilized through organizational capabilities that translate them into coordinated actions. Patient agility appears to fulfil this role by connecting the strategic orchestration of digital resources with the operational processes involved in identifying and responding to patient needs.

From a dynamic capabilities perspective, digital transformational leadership can therefore be conceptualized as an antecedent of higher-order organizational capabilities, whereas patient agility represents a more proximal capability associated directly with care processes. This distinction highlights the multilevel nature of value creation in digitally transforming organizations: strategic-level leadership must be translated into operational-level capabilities before it can be reflected in observable organizational outcomes.

This interpretation is consistent with the literature on digital transformation in healthcare, which suggests that technologies and leadership generate value only when they are embedded in organizational processes and translated into concrete practices.^26^,^45^

The findings therefore position patient agility as a critical link between the strategic level of leadership and the operational level of care coordination. These results can also be interpreted through the lens of socio-technical systems theory. Effective care coordination requires alignment between technology, people, workflows, communication, and organizational rules.^12^

From this perspective, digital transformational leadership may support care coordination indirectly by facilitating the alignment of the socio-technical system and creating conditions conducive to coordinated action. Patient agility, in turn, reflects the organizational capacity to enact this alignment by detecting patient-related signals and responding to them through integrated care processes. Leadership thus supports system-level alignment, while patient agility helps translate that alignment into everyday clinical and organizational practice. This is particularly relevant in primary healthcare settings, where coordination depends on the continuous integration of information, communication among professionals, and flexible management of care pathways for patients with multimorbidity.

The findings also contribute to the literature on coordinated care and multimorbidity management. Previous research has often focused on the role of technology, interoperability, or formal integration mechanisms, while relatively less attention has been paid to organizational capabilities that enable the effective use of these resources.

The results suggest that in primary healthcare organizations responsible for coordinating long-term care for patients with complex conditions, the key factor is not digitalization itself, but the organization’s capability to recognize patient needs and respond to them in a timely and coordinated manner. Overall, the findings indicate that effective care coordination is associated not only with digital infrastructure and leadership but also with the adaptive organizational capabilities through which digital resources are interpreted, mobilized, and incorporated into care processes. This interpretation remains provisional and should be examined further using longitudinal, multi-source, and patient-level data.

## Conclusion

This study advances the understanding of how digital transformational leadership is associated with coordinated care in primary healthcare organizations by identifying patient agility as a potential statistical mediator. The findings indicate that digital transformational leadership has a relatively weak direct association with coordinated care, whereas its overall association with coordinated care is largely explained statistically by patient agility, an organizational capability that enables healthcare providers to sense and respond to evolving patient needs. These findings provide a more nuanced explanation of how leadership may contribute to healthcare outcomes by highlighting the importance of capability development alongside technological change. From a theoretical perspective, the findings contribute to the integration of Dynamic Capabilities Theory, the Resource-Based View, and Socio-Technical Systems Theory in the context of healthcare. They support the view that digital transformational leadership functions as an antecedent of dynamic capabilities by strengthening organizational routines associated with sensing and responding to patient needs.^4^,^10^,^18^ Patient agility emerges as a concrete manifestation of these capabilities, linking strategic leadership with coordinated care. Furthermore, the relatively weak direct relationship between leadership and coordinated care is consistent with the Resource-Based View, suggesting that leadership alone is unlikely to improve organizational outcomes without complementary capabilities.^11^ The findings also extend Socio-Technical Systems Theory by indicating that aligning technological and organizational elements may be necessary but not sufficient to improve coordinated care unless organizations develop the capability to act upon patient-related information in an adaptive manner. From a practical perspective, the findings suggest that improving coordinated care requires more than the adoption of digital technologies alone. While digital transformation provides the necessary technological foundation, its benefits are unlikely to be realized without leadership capable of developing organizational capabilities that enhance patient agility. Healthcare managers should therefore view digital transformational leadership not merely as a driver of technology implementation but as a mechanism for fostering adaptive organizational processes that enable timely responses to patients’ evolving needs. These findings further suggest that digital technologies create value only when they are effectively embedded within organizational routines, clinical workflows, and interprofessional collaboration. Accordingly, successful digital transformation should be understood as an organizational rather than purely technological process. Despite these contributions, several limitations should be acknowledged. First, the use of cross-sectional data limits the ability to draw strong causal inferences. Although the hypothesized relationships were supported statistically, the findings should be interpreted as evidence of associations rather than definitive causal mechanisms. Future research should employ longitudinal, experimental, or time-lagged designs to establish temporal ordering and better capture the evolution of leadership and capability development over time. Second, the reliance on survey-based measures collected simultaneously from a single respondent source introduces the potential risk of common method bias. Although Harman’s single-factor test did not indicate that a single factor dominated the data, the common latent factor analysis suggested the presence of some shared method-related variance. After controlling for this factor, the principal structural relationships remained positive and statistically significant; however, the explained variance in coordinated care decreased from 78.7% to 49.4%. Accordingly, the comparatively high R^2^ observed in the baseline model should be interpreted cautiously, as common source variance may have contributed to its magnitude. Subsequent studies are encouraged to incorporate multi-source data, including objective performance indicators, independently assessed organizational measures, and patient-reported outcomes, to enhance the robustness of the findings. Third, participants were recruited through commercial non-probability online panels, which may introduce self-selection and coverage bias and may limit the representativeness of the sample. Although country-specific quotas, eligibility screening, and quality-control procedures were applied, these measures cannot fully eliminate potential panel-related biases. Moreover, because recruitment and survey invitation distribution were managed by the panel provider, invitation-level disposition data were not available to the research team, and an invitation-based response rate could not be calculated. Future studies could address these limitations by using probability-based sampling, alternative recruitment strategies, or multiple sampling sources. Fourth, the focus on primary healthcare organizations may limit the generalizability of the results. Future research should examine whether the observed relationships hold across different healthcare contexts, such as hospitals or integrated care systems. Additionally, the study adopts an organizational-level perspective and does not directly assess patient-level outcomes, such as satisfaction, adherence, or clinical outcomes. Exploring whether and under what conditions patient agility is associated with measurable improvements in patient experiences and health outcomes represents an important avenue for future research. Although the mediating role of patient agility was statistically supported, it is likely that additional mediating and moderating mechanisms, such as organizational culture, digital maturity, and inter-organizational collaboration, may also shape the relationship between leadership and coordinated care.^16^,^46^ Investigating these factors would provide a more comprehensive understanding of digital transformation processes in healthcare. Finally, future studies could examine the microfoundations of patient agility, including the role of individual competencies, learning processes, and digital infrastructures in shaping sensing and responding capabilities. Overall, the findings suggest that patient agility represents a promising explanatory mechanism through which digital transformational leadership may be associated with coordinated care. Nevertheless, confirming this mechanism will require longitudinal and intervention-based studies capable of establishing temporal relationships and strengthening causal inference.
